# Mental health of forced migrants recently granted leave to remain in the United Kingdom

**DOI:** 10.1177/0020764020939610

**Published:** 2020-07-20

**Authors:** Sophie Walker, Martha von Werthern, Francesca Brady, Cornelius Katona

**Affiliations:** 1 Helen Bamber Foundation, London, UK; 2 Psychiatry, University of Oxford, UK; 3 Department of Mental Health Sciences, UCL, London, UK; 4 Woodfield Trauma Service, Central and North West London Mental Health NHS Trust, UK; 5 Division of Psychiatry, University College London, London, UK

**Keywords:** Refugee, transition period, mental health, forced migration

## Abstract

**Background:**

Asylum seekers who are granted leave to remain in the United Kingdom are required to make a rapid transition to housing and welfare benefits. The challenges facing new refugees during this ‘transition period’ can affect their mental health, but this has not been quantified.

**Aims:**

To assess the impact of the transition period on new refugees’ mental health in the 12 months after being granted leave to remain in the United Kingdom.

**Method:**

A longitudinal survey design was used to measure the mental health of 30 newly recognised refugees at monthly intervals in the first 6 months and again at 1 year after receiving leave to remain in the United Kingdom. There were five outcome measures for symptoms of anxiety, depression, distress, post-traumatic stress disorder (PTSD), post-migration living difficulties (PMLD) and a life events calendar to record key changes in housing and welfare.

**Results:**

The results showed that the trajectory of scores across all measures fluctuates, but overall they all improve from baseline to Month 12. Scores for depression and PMLD showed significant improvement at Month 5, and scores for anxiety, depression, distress and PMLD showed significant improvement at Month 12. PTSD scores did not show significant improvement at any month. In months with a high number of stressful life events, participants had worse PMLD and PTSD scores.

**Conclusion:**

Overall improvement in mental health could partly be explained by the stability of being granted leave to remain in the United Kingdom, but may also be due to the high level of practical support these participants received. Recommendations are made for those working with clients during the transition period.

## Background

According to the 1951 United Nations Convention, a refugee is a person who has been forced to flee their country of origin and is not willing or able to return to it due to ‘a well-founded fear of being persecuted for reasons of race, religion, nationality, membership of a particular social group or political opinion’. In the United Kingdom, an individual who has applied for asylum becomes a refugee when the government agrees that she or he meets this definition.

In 2018, 29,380 asylum applications were made in the United Kingdom, with 32.5% of the applicants granted a form of long-term leave to remain by the Home Office or independent tribunal (Refugee Council, 2019). For 41.6% of applicants, the wait for an initial decision was more than 6 months, with some waiting years or undergoing lengthy appeals (Home Office, 2018).

Asylum seekers who are granted leave to remain in the United Kingdom are issued with two identity documents: a Biometric Residence Permit (BRP) and a National Insurance Number. These are needed to apply for employment or welfare benefits and for social housing. Once they have received their BRP, any ‘Asylum Support’ subsistence payments or accommodation provision from the Home Office ceases after 28 days. The shift from being an asylum seeker to having leave to remain in the United Kingdom during and after this 28-day period is referred to herein as the transition period.

The difficulties faced by newly recognised refugees during this period have begun to be documented by the charity sector in recent years. However, there are no published studies on the impact of these challenges on individuals’ mental health and the trajectory of this impact over time after gaining leave to remain.

### Challenges during the transition period

A recent report published by the British Red Cross (2018) suggests that 28 days is too short a time for applications to the services required by newly recognised refugees (such as welfare benefits) to be processed. Receipt of benefits may also be delayed because of barriers to setting up bank accounts, such as being unable to produce accepted forms of ID or proof of address. A report by the Refugee Council based on interviews with 11 new refugees found that during the 28-day transition period, only one person had secured welfare benefits and none of the participants had secured work. It took four participants over 6 months to find a job (Basedow & Doyle, 2016). Government subsistence payments are not sufficient to save for the deposit required to access private rented accommodation, and Integration Loans to cover this deposit cannot be applied for before the Home Office accommodation contract is terminated (i.e., at the end of the 28 days).

As a result of such issues, refugees are at significant risk of facing homelessness and destitution during their transition period and are forced to rely on the generosity of friends and support of faith groups and charities in order to eat and find shelter (Doyle, 2014). Another report from the Red Cross which studied 14 newly recognised refugees found that they had all experienced some form of destitution (defined as not having financial support, adequate accommodation or both) during the transition period (Carnet et al., 2014). A recent follow-up report by The No Accommodation Network (NACCOM, 2019) found that the percentage of newly recognised refugees accessing their night shelters within 6 months of being granted leave to remain rose from 21% in 2017/2018 to 36% in 2018/2019; some were still homeless a year after being granted leave to remain.

Organisations such as the Red Cross and NACCOM have highlighted the need for structural changes to services and for more advice and support for new refugees to avoid destitution and improve the success of the transition period. However, no research to date has examined the impact of such transition-related stress on the mental health of refugees.

### Refugee mental health

Refugees experience diverse stressors that accumulate over the pre-flight, flight, exile and resettlement periods (Porter & Haslam, 2005); a knowledge of these stressors aids an understanding of the context within which many refugees navigate the transition period.

Prevalence rates of mental disorders in settled war refugees vary greatly due to both clinical and methodological factors, with depression ranging from 2.3% to 80%, unspecified anxiety disorder from 20.3% to 88% and PTSD from 4.4% to 86%, although prevalence estimates are typically in the range of 20% and above (Bogic et al., 2015). Prevalence of PTSD is estimated at 9% in the general refugee population (Fazel, Wheeler & Danesh, 2005), but is as high as 69%–92% in refugee survivors of torture (Moisander & Edston, 2003).

Post-migration factors, in particular socio-economic status, are more powerful predictors of depression, anxiety and substance use disorder than pre- or peri-migration factors (Bogic et al., 2012). These post-migration factors include adverse living conditions, employment and financial concerns, language barriers, family separation, illness, lack of social support and acclimatising to a new culture (Laban et al., 2005; Li et al., 2016; Schweitzer et al., 2011). In addition, experiences of detention (von Werthern et al., 2018) and of threat of removal from the country of exile (Reesp, 2003) are associated with more negative mental health outcomes.

A number of systematic reviews have found that socio-demographic characteristics may also moderate mental health outcomes in refugee populations. When limited to diagnosed mental disorders, prevalence rates of depression, unspecified anxiety disorder and PTSD were associated only with gender and country of origin (Bogic et al., 2015). When defining psychological health more broadly to include poor daily functioning and stress, female gender, older age, not being married and lower education level were also associated with worse psychological health (Roberts & Browne, 2011).

Many refugees will not have received any support or treatment for their mental health issues in their host country (Doctors of the World, 2017). A lack of specialist services, interpreters, illiteracy, stigma, fear of disclosure, an unfamiliar system and access issues all constitute major barriers preventing new refugees from receiving the health care they need (All Party Parliamentary Group, 2017).

### Mental health in the transition period

The further potential adversity, pressure and unpredictability that the transition period stressors impose on new refugees create a clinically high-risk situation for those with mental health vulnerabilities. This is due to the short time frame in which such significant financial and housing arrangements must be made, in conjunction with the lack of ease and transparency with which these issues can pragmatically be addressed.

The Refugee Council’s 2016 report highlights the impact of stress and instability of the transition period on refugees’ mental health. All participants interviewed reported an increase in anxiety and depression symptoms (both clinically diagnosed and self-reported), with some also reporting suicidal thoughts (Basedow & Doyle, 2016). These symptoms have been shown to be exacerbated by isolation, a lack of social connections and purpose in the community due to not having a secure job or home, and experiences of hostility and racism in the host country (Burnett & Peel, 2001). Such experiences may trigger distressing memories of past traumatic experiences and may also exacerbate pre-existing mental health difficulties, such as PTSD, anxiety, depression or other problems such as loss and bereavement.

Many asylum seekers expect that arrival in the United Kingdom and then gaining leave to remain will signal an ‘end’ to their difficulties. Unfortunately, this is frequently not the case; the psychological impact of this realisation can be significant, especially in those who have not ‘emotionally processed many of their experiences, losses and changes’ (British Psychological Society (BPS), 2018). There remains a need to explore the impact of the transition period on the mental health of new refugees, by measuring psychological outcomes longitudinally to investigate the nature and timing of the difficulties experienced, and thus what kind of support may be required. This study focuses on this impact in the United Kingdom due to our research having taken place there and our familiarity with the UK context. However, other countries may learn that the design of this study and the nature of these difficulties may apply to their own populations, despite the global variation in immigration systems.

### Aims of research

This study focused on the experiences of adults with a history of forced migration (i.e., of having to flee their country due to fears of persecution or other reasons, or survivors of trafficking). It aimed to assess the impact of the transition period on the new refugees’ mental health in the 12 months after being granted leave to remain in the United Kingdom. It was hypothesised that overall participants would improve, but that any deterioration of mental health during this period would be correlated with key negative life events and upheaval related to transition.

## Method

### Setting

The study took place at a London-based charity delivering specialist care for survivors of human rights abuses (including war trauma, torture, human trafficking, domestic or gender-based violence). Individual clients are offered a ‘Model of Integrated Care’ incorporating therapeutic care and health care advice, as well as legal and welfare support to address their needs.

### Participants

The study sample consisted of a consecutive cohort of all eligible clients of the charity who had been granted any type of leave to remain in the United Kingdom (*N* = 30).

Twenty-three participants (79%) had been granted Refugee Status (RS), and one (3%) had received Humanitarian Protection (HP) both of which permit leave to remain in the United Kingdom for an initial period, which is usually 5 years. Five participants (17%) received Discretionary Leave to Remain, which is of variable duration of up to 2.5 years and is granted to individuals not qualifying for RS or HP who have other strong grounds for staying in the United Kingdom.

There were two exclusion criteria: (1) duration of leave shorter than 12 months, and (2) individuals who were at high risk, for example, where there was evidence of high risk of suicide or active psychosis.

Recruitment took place between April 2016 and November 2017. A total of 30 eligible clients agreed to participate and were included in the study. Of those approached, 91% agreed to participate, with the remaining 9% (*n* = 3) declining due to the stress of the approaching transition. Out of 30 participants enrolled, one dropped out after Month 1. Twenty-nine participants completed Month 5, and 28 completed Month 12. This provided sufficient and on-time data for the analysis of 1,362 (82.5%) responses. At each month, the mean number of missing data points was 5.

Table 1 presents the socio-demographic information collected at baseline (Month 0) for the total sample (*N* = 29). Twenty-two (76%) participants received therapeutic support before or during the study, and 16 (53%) were on psychiatric medication, of whom 10 (33%) were on antidepressants and 6 (20%) on antipsychotics.

**Table 1.** Baseline socio-demographic variables of sample (*N* = 29).

Characteristic	*n* (%)
Age	Mean = 36; *SD* = 9.17
	Range = 18–63
Gender	
Female	17 (58.6)
Male	12 (41.4)
Ethnic group	
African	14 (48.3)
Arab	3 (10.3)
European	4 (13.8)
South Asian	8 (27.6)
Marital status	
Single	17 (58.6)
Married	7 (24.1)
Divorced/Widowed	3 (10.3)
Separated	2 (6.9)
Number of children in the United Kingdom/Outside the United Kingdom	
None	12 (41.4) / 11 (37.9)
1	5 (17.2) / 6 (20.7)
2	3 (10.3) / 4 (13.8)
Years of education	
>5	9 (31)
<5	6 (20.7)
<10	14 (48.3)
Level of English language	
Basic	7 (24.1)
Conversational	6 (20.7)
Fluent	12 (41.4)
Type of support at baseline	
Housing benefit (ESA, JSA, Child)	7 (24.1)
NASS Section 5	9 (31)
NASS Section 4	2 (6.9)
Nothing (staying with family/friends)	7 (24.1)

*SD*: standard deviation; ESA: employment and support allowance; JSA: jobseeker’s allowance; NASS: National Asylum Support Service.

### Procedure

Staff members who were aware of changes in an individual’s asylum seeker status identified potential participants. An advertisement was placed in the charity’s reception area so that individuals could also self-refer into the study.

Potential participants were informed about the study’s aims and procedures, and written informed consent was obtained from all subjects. Once participants had received their BRP, they completed a set of baseline measures (Month 0). These were repeated on a monthly basis for a period of 5 months, with follow-up data collected 12 months after baseline, making a total of seven time-points. Participants who used a professional interpreter as part of their routine engagement with the charity used the same interpreter to complete the study measures. Overall, four interpreters were used to read questions for six participants.

### Ethical considerations

The authors assert that all procedures contributing to this work comply with the ethical standards of the relevant national and institutional committees on human experimentation and with the Helsinki Declaration of 1975, as revised in 2008. All procedures involving human subjects were approved by the University College London Ethics Committee (8133/001).

### Measures

#### Generalised Anxiety Disorder-7

The seven-item Generalised Anxiety Disorder Scale (GAD-7; Spitzer et al., 2006) is a brief rating scale used to measure the severity of anxiety symptoms experienced in the prior 2 weeks. Total score categories are as follows: 5–9 (mild), 10–14 (moderate) and 15–21 (severe). The GAD-7 has good sensitivity (89%) and specificity (82%). It has excellent internal consistency (α= .92), along with good test–retest validity (intra-class correlation = .83) and procedural validity (intra-class correlation = .83).

#### Patient Health Questionnaire-9

The nine-item Patient Health Questionnaire (PHQ-9; Kroenke et al., 2001) is a brief rating scale used to measure the severity of depression symptoms experienced in the prior 2 weeks. Total score categories are as follows: 5–9 (mild), 10–14 (moderate), 15–19 (moderately severe) and 20–27 (severe). The PHQ-9 has good sensitivity (88%) and specificity (88%) for diagnosing major depression, as well as excellent internal reliability (α = .89/.86 across studies) and test–retest reliability.

#### Clinical Outcomes in Routine Evaluation-10

The 10-item Clinical Outcomes in Routine Evaluation-10 (CORE-10; Barkham et al., 2006) is a brief rating scale used to measure overall mental distress in the past week. Total score categories are as follows: 0–5.9 (normal), 6.2–9.7 (low distress), 10–14.7 (mild distress), 15–19.7 (moderate distress), 20–24.7 (moderate/severe distress) and >25 (severe distress). It has good internal reliability (α = .90), excellent sensitivity (.92) and good specificity (.72).

#### PTSD Checklist for *DSM*-5

The PTSD Checklist for *DSM*-5 (PCL-5; Weathers et al., 2013) is a 20-item self-report measure that assesses the presence and severity of PTSD symptoms in the past month. Items on the PCL-5 correspond with the *Diagnostic and Statistical Manual of Mental Disorders*, Fifth Edition (*DSM*-5) criteria for PTSD. Respondents rate each item using a 5-point Likert-type scale from 0 to 4, providing a total severity score (range = 0–80). The higher the score, the greater the symptom severity, with a total score of 33 or above suggesting the patient may meet the threshold for a diagnosis of PTSD. However, a formal diagnosis of PTSD can only be made by clinical interview.^25^ The PCL-5 has strong internal consistency (α = .94), test–retest reliability (*r* = .82), and convergent (*r*s = .74–.85) and discriminant (*r*s = .31–.60) validity (Blevins et al., 2015).

#### Post-Migration Living Difficulties Checklist

The Post-Migration Living Difficulties (PMLD; Silove, 1999) Checklist is a 22-item self-evaluated measure used to assess current life stressors of asylum seekers in the past month using a 5-point Likert-type scale from *no problem* to *very serious problem*. Principal component analyses yielded five factors accounting for 69.8% of the variance of the 23 items: refugee determination process; health, welfare and asylum problems; family concerns; general adaptation stressors; and social and cultural isolation. In addition, the stress measured by PMLD items is independent of past trauma in predicting psychiatric outcomes such as PTSD.

#### Life events calendar

A life events calendar was developed specifically for use in this study (see Appendix 1). Participants were asked to record monthly changes in the following subcategories: housing, finance, welfare, education and employment. At each time-point, they were asked to rate the number of life events in each category and rate the impact these events had on them using a 5-point Likert-type scale (1 = *very good*, 5 = *very bad*). The overall monthly score was the mean average score of all five subcategories, rounded to the nearest whole number.

### Data analysis

A linear mixed model for repeated measures over time was used to analyse the outcome measures at each time-point compared with baseline scores. This was because the data are nested within person (i.e., not independent). The model also prevents listwise deletion due to missing data and can handle subjects measured incompletely or at different time-points.

We looked at the interactions between time and the life events calendar on each of the outcome measures and also tested whether gender, age, area of origin, marital status and education level were associated with the outcome measures, based on previous associations in the literature (Bogic et al., 2015; Roberts & Browne, 2011).

SPSS statistical software, Version 25.0 (International Business Machines Corporation, 2017) was used for data analysis. We computed descriptive statistics by using the mean and standard deviation for each of the six outcome measures. We constructed models to predict change in scores over the 12 months after receiving leave to remain. Normality of residuals does not affect the parameter estimates in multi-level models (Gelman & Hill, 2006); therefore, this was not tested. Subjects were treated as random effects, and time was treated as a categorical fixed effect; SPSS automatically dummy-codes these variables, with the reference group (Month 0 – Baseline) coming last. There were five continuous outcome measures: GAD-7 (anxiety), PHQ-9 (depression), CORE (distress), PCL-5 (post-traumatic stress) and PMLD. All items from these measures were weighted equally in a parametric mixed model. The sixth outcome measure was the categorical life events calendar, on which a non-parametric model was run.

## Results

Table 2 presents the means and standard deviations of each outcome measure at each of the seven time-points (Months 0, 1, 2, 3, 4, 5 and 12). The results of the linear mixed model for each of the five continuous outcome measures are shown in Table 3.

**Table 2.** The means and standard deviations of each outcome measure at each time-point.

	Month 0	Month 1	Month 2	Month 3	Month 4	Month 5	Month 12
GAD-7	14.37 (1.18)	13.06 (1.03)	12.95 (0.98)	13.47 (1.10)	12.34 (1.05)	12.07 (1.14)	6.62 (1.58)
PHQ-9	16.82 (1.33)	15.70 (1.19)	14.43 (1.25)	14.77 (1.13)	14.05 (1.15)	13.46 (1.22)	7.21 (1.87)
CORE-10	21.91 (1.45)	21.18 (1.32)	21.14 (1.45)	20.12 (1.27)	20.18 (1.43)	18.98 (1.53)	10.70 (2.33)
PCL-5	49.07 (3.97)	43.93 (4.01)	45.90 (3.70)	43.47 (3.38)	42.98 (3.71)	40.97 (3.86)	38.80 (4.10)
PMLD	59.41 (4.19)	51.59 (3.93)	44.54 (4.65)	45.27 (3.83)	41.51 (3.21)	35.61 (3.48)	30.94 (4.76)
LEC	3.74 (0.15)	3.79 (0.16)	3.97 (0.12)	3.74 (0.16)	4.04 (0.11)	3.74 (0.13)	2.87 (0.28)

GAD-7: Generalised Anxiety Disorder-7; PHQ-9: Patient Health Questionnaire-9; CORE-10: Clinical Outcomes in Routine Evaluation-10; PCL-5: PTSD Checklist for *DSM*-5; PMLD: Post-Migration Living Difficulties; LEC: life events calendar.

**Table 3.** The fixed estimates of the parametric mixed linear model (confidence interval coefficients 95%) for each outcome measure.

	Month 0	Month 1	Month 2	Month 3	Month 4	Month 5	Month 12
GAD-7	14.37 (11.99 to 16.76)	−1.31 (−3.25 to 0.63)	−1.42 (−3.25 to 0.41)	−0.90 (−2.97 to 1.17)	−2.03 (−4.01 to −0.05)	−2.30 (−4.47 to −0.14)	−7.75** (−10.88 to −4.62)
PHQ-9	16.82 (14.15 to 19.49)	−1.12 (−3.34 to 1.09)	−2.39 (−4.74 to −0.04)	−2.05 (−4.15 to 0.06)	−2.28 (−4.92 to −0.62)	−3.36* (−5.63 to −1.09)	−9.61** (−13.30 to −5.91)
CORE-10	21.91 (18.97 to 24.86)	−0.74 (−3.25 to 1.78)	−0.77 (−3.56 to 2.01)	−1.79 (−4.19 to 0.61)	−1.74 (−4.47 to 1.00)	−2.94 (−5.88 to 0.00)	−11.21** (−15.89 to −6.53)
PCL-5	49.07 (41.05 to 57.09)	−5.14 (−12.01 to 1.74)	−3.17 (−9.32 to 2.98)	−5.59 (−10.96 to −0.23)	−6.09 (−12.25 to −0.07)	−8.10 (−14.63 to −1.58)	−10.27 (−17.43 to −3.11)
PMLD	59.41 (50.99 to 67.84)	−7.82 (−16.44 to 0.80)	−14.88* (−24.84 to −4.92)	−14.14* (−22.52 to −5.76)	−17.91** (25.24 to −10.57)	−23.81** (−31.59 to −16.02)	−28.47** (−38.77 to −18.17)

GAD-7: Generalised Anxiety Disorder-7; PHQ: Patient Health Questionnaire-9; CORE-10: Clinical Outcomes in Routine Evaluation-10; PCL-5: PTSD Checklist for *DSM*-5; PMLD: Post-Migration Living Difficulties.

* *p* < .001; **p < .0001.

The mean scores in Table 2 show that the linear trajectory of scores across all measures over time fluctuates, but overall they all improve from baseline (Month 0) to Month 12. The estimates of fixed effects shown in Table 3 indicated that most scores improved significantly towards the end of the study at Months 5 and 12. GAD-7 scores for anxiety showed linear improvement during the study with deviations at Months 3 and 5, and a significant improvement compared to baseline at Month 12 (*b* = −7.75, *p* = .0001). PHQ-9 scores for depression also showed linear improvement during the study with deviations at Months 3 and 5, and a significant improvement compared to baseline at Month 5 (*b* = −3.36, *p* = .005) and Month 12 (*b* = −9.61, *p* = .001). CORE-10 scores for distress showed linear improvement during the study with a deviation at Month 5 and a significant improvement compared to baseline at Month 12 (*b* = –2.94, *p* = .050). PSSI scores for post-traumatic stress symptoms showed overall linear improvement by Month 12, but with no significant improvement at any month. PMLD scores for general functioning relating to post-migration living difficulties were significantly improved compared to baseline at Months 2, 3, 4, 5 (*b* = −23.81, *p* = .0001) and 12 (*b* = −28.47, *p* = .0001).

A non-parametric linear mixed model was used to look at the interactions between time and the life events calendar on each of the five mental health outcome measures. A significant association was found between time and the life events calendar on PCL-5 (3.06 (1.14 to 4.98), 0.002) and between time and the life events calendar on PMLD (5.39 (2.25 to 8.53), 0.001). There was, however, no significant interaction between time and the life events calendar on GAD-7 (0.92 (0.12–0.73), 0.024), PHQ-9 (0.36 (–0.46 to 1.19), 0.380) and CORE-10 (0.59 (–0.48 to 1.67), 0.277) scores. There was no significant association found using the linear mixed model on the socio-demographic characteristics of gender, age, area of origin, marital status or education level and any of the six outcome measures.

## Discussion

The aim of this study was to investigate the impact of the transition period on the mental health of newly recognised refugees. The scores for anxiety, depression, distress and PMLD showed linear improvement overall during the 12-month study, with scores for depression and PMLD showing significant improvement at Month 5 and scores for anxiety, depression, distress and PMLD showing significant improvement at Month 12.

Scores for post-traumatic stress symptoms also showed linear improvement throughout the study but did not show significant improvement at any month. This supports previous evidence that improvement in the stability of accommodation and ability to find work predicts improvement in mood and anxiety symptoms, but not necessarily PTSD symptoms (Roberts & Browne, 2011). This may be because dysfunctional cognitive appraisals of trauma in PTSD are automatic and relatively fixed, whereas worry mechanisms in general anxiety are strategic processes (Riskind, 2005) and can be moderated by social factors.

Months with a high life events calendar score (indicating significant change or upheaval) were associated with significantly worse PMLD and post-traumatic stress symptoms. This is surprising since recent adverse life events would be expected to be associated with greater worsening of anxiety or depression symptoms than of PTSD symptoms (Bogic et al., 2012). Further research is needed to establish the mechanisms whereby adverse life events exacerbated PTSD symptoms – perhaps by acting as reminders of past trauma.

There was no significant association between any of the socio-demographic variables of gender, age, area of origin, marital status, or education level and the outcome measures. This may reflect the small sample size for each variable and consequent lack of statistical power. Future studies using bigger sample sizes may be able to explore whether these associations do exist.

The overall improvement in mood and well-being observed in this study could be explained broadly by the following two explanations – which are not mutually exclusive. First, the stability associated with being granted leave to remain, sometimes after prolonged uncertainty about the future, may help to start improving mood scores over time. A qualitative study examining participants’ perceptions of the transition period that ran parallel to this study supported these findings that gaining status represents gaining control and stability over one’s life (Rowley, Morant & Katona, 2019).

The second explanation draws on the uniqueness of the sample in this study. All participants were recruited from a London-based charity, where they received a high level of support for complex and severe psychological, medical, legal, welfare and housing difficulties. The extra practical advice and signposting may make navigating the challenges of the transition period somewhat easier, but it is unclear whether it was specific aspects of the support (such as psychological therapy or individual support with advocacy on welfare issues) that explained the improvement in mental health or whether it was due to the holistic approach of support in all areas.

Indeed, the mechanisms by which support works for different groups of refugees (potentially with different needs), in addition to the complexity of support, support-seeking behaviours and appraisal of support received, have been overlooked in research to date (Leduc & Proulx, 2004). Further research incorporating a comparison group of individuals receiving no support during transition would determine whether the support itself contributed to the improvement.

### Limitations

Conducting research with refugee populations can be challenging and may encounter missing data, attrition and participant difficulty with engagement. Indeed, this sample may suffer from self-selection bias given there were three clients approached who declined to take part due to transition-related stress.

The small size and specific characteristics of the participants in this study limit the generalisability of the findings. Participants were especially vulnerable and receiving specialist support from the charity; further research is needed to represent a larger, more diverse sample of refugees in the United Kingdom and also to represent refugees globally. It could benefit from a control group of individuals still waiting for their asylum decision to distinguish between post-migration and more transition-specific mental health issues.

A further limitation was the timing of the baseline visit which only took place after the BRP was received. This was chosen because it is when the 28-day transition period starts. However, this meant that participants were not assessed during the period between receiving status and receiving BRP. Future studies should design the baseline visit for as soon as status is granted.

The self-report measures used in this study have not been explicitly validated for use in refugee populations with formal psychiatric diagnoses. The lack of formal translations or validated measures in other languages may have led to potential inconsistencies between interpreters, leading to bias in the findings.

### Clinical recommendations

In addition to the current BPS guidelines (BPS, 2018) for professionals working with asylum seekers and refugees in general, the authors propose the following recommendations for the transition period. Although they are derived from our UK experience, we consider their principles to be applicable globally:

- Professionals should be aware that although newly recognised refugees’ mental health may overall improve in the 12 months after receiving leave to remain, it is also liable to fluctuate during this period. Mood and anxiety deterioration may still occur, especially in the first 6 months after gaining status when living instability may still be an issue, and clients may need additional support and risk assessment at these times.
- Individuals with PTSD may need additional support during the transition period, with a particular focus on triggers occurring as a result of new changes relating to transition.
- Robust referral pathways to support individuals who do not improve after gaining leave to remain should be in place to minimise disruption in their clinical care.

This study is the first collection of data of its kind in the United Kingdom and is therefore an important starting point for future research in this area. However, suggestions have been made as to how the design of future studies could be improved to provide data that can be well utilised by service providers and policy makers in improving the experience of the transition period in the United Kingdom. The authors have also made clinical recommendations for those working with individuals who gain leave to remain in the United Kingdom.

## Appendix 1

### Life events calendar

*Instructions*: please complete this grid to tell us in the past month:

1. How many life events happened in each of the five life events categories below?
  (E.g., having your Asylum Support payments stopped would be 1 event)
2. What was the impact of the life event/s on you?
  (E.g., if the Asylum Support stopping had a bad effect on you, put a cross under the column ‘Bad’).

Here’s an example of how to complete the grid:

**Table:**

	(1)	(2)				
	How many events occurred?	Very good	Good	Neither good nor bad	Bad	Very bad
*Financial* (benefits)	2				X	

In the past month:

**Table:**

	(1)	(2)				
	How many events occurred?	Very good	Good	Neither good nor bad	Bad	Very bad
*Housing* (homeless/energy/access)						
*Financial* (benefits)						
*Welfare* (GP/children/dependents)						
*Education* (ESOL/college)						
*Employment* (engagement)						
If you would like to tell us any more detail about the life events that have happened in the past month, please do below:						

ESOL: English as a Second or Other Language; GP: general practitioner.
